# 1226. Differences in diagnosis and procedure codes used among 5 Veterans Affairs telehealth antibiotic stewardship services

**DOI:** 10.1093/ofid/ofad500.1066

**Published:** 2023-11-27

**Authors:** Sara A Abdelrahim, Brigid Wilson, Amanda Vivo, Tammy Walkner, Tola M Ewers, Daniel J Livorsi, Christopher J Crnich, Rabeeya Sabzwari, Andrew S Webster, Lauren H Epstein, Amelia Milner, Robin L Jump, Charlesnika T Evans, Taissa A Bej

**Affiliations:** VA Northeast Ohio Healthcare System, Cleveland, Ohio; VA Northeast Ohio Healthcare System, Cleveland, Ohio; CINCCH at Hines VA, Hines, IL; Iowa City Veterans Healthcare System, Iowa City, Iowa; University of Wisconsin-Madison, Madison, Wisconsin; University of Iowa Carver College of Medicine, Iowa City, Iowa; University of Wisconsin School of Medicine and Public Health, Madison, Wisconsin; Edward Hines, Jr. VA Hospital, Hines, Illinois; Atlanta VA Medical Center, Decatur, Georgia; Emory University School of Medicine, Atlanta, GA; VA Northeast Ohio Healthcare System, Cleveland, Ohio; University of Pittsburgh School of Medicine, Cleveland, Ohio; Northwestern University and VA, Hines, Illinois; Louis Stokes Cleveland VA Medical Center, Cleveland, Ohio

## Abstract

**Background:**

Telehealth offers an opportunity for rural healthcare settings to connect with infectious disease (ID) experts to augment clinical care of people with suspected infections and to support antimicrobial stewardship efforts. We are currently disseminating a project that uses Veterans Affairs (VA) telehealth systems to connect multidisciplinary teams from rural VA medical centers with ID experts at geographically distant locations to form Videoconference Antimicrobial Stewardship Teams (VASTs). Here, we used diagnosis and procedure codes to describe preliminary outcomes from 5 VASTs, assessing the time used for each consult as well as the infectious and other clinical syndromes discussed.

**Methods:**

We extracted Current Procedural Terminology (CPT) and International Classification of Diseases, Tenth Revision (ICD-10) codes associated with clinical encounters from January 2022 – April 2023 for 5 VASTs. CPT codes indicated the time spent by the ID expert. ICD-10 codes were summarized into the following broad categories: infections, pathogens, other clinical diagnoses, signs and symptoms, treatments, and administrative codes.

**Results:**

A total of 315 cases were discussed across all 5 sites, with most cases taking < 30 minutes to complete (210; 67%; **Figure 1**). The cases yielded 600 ICD-10 codes, of which 219 (36%) described an infection and 165 (27%) described other medical diagnoses (**Figure 2**). The number of ICD-10 codes per consult ranged from 1.0 (Sites A and C) to 3.5 (Site D). Overall, the most common infections discussed pertained to the respiratory tract (57/219, 26%) and skin and soft tissue (53/219, 24%); the most common pathogen code was for *Enterococcus* spp. (10/59, 17%) (**Figure 3**).

**Figure 1:**
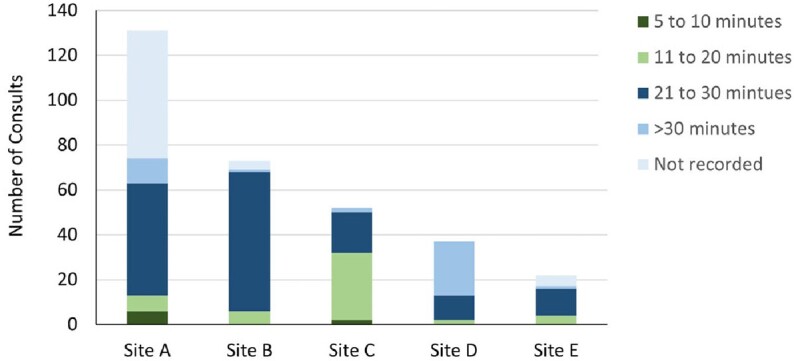
Length of Time per Consult

**Figure 2:**
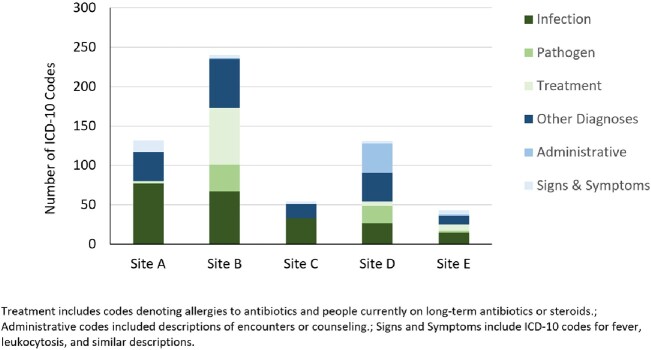
Types of ICD-10 Codes

**Figure 3:**
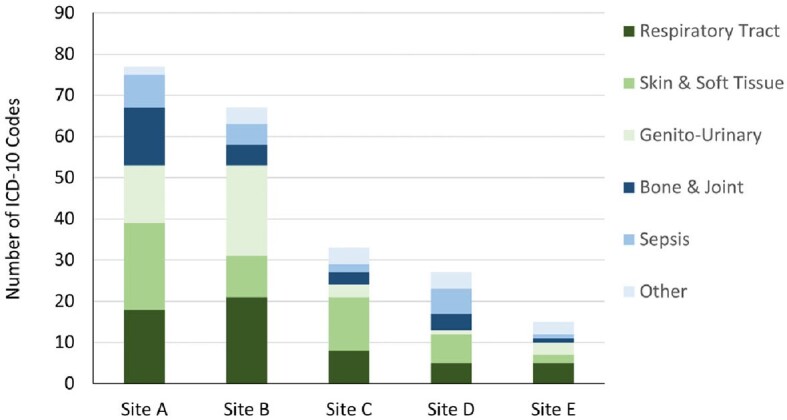
ICD-10 Codes for Infections

**Conclusion:**

Our results reveal notable differences in the diagnosis and procedure codes used across the 5 VASTs. In general, the VASTs address a similar array of infectious disease syndromes, suggesting that the observed differences may relate to how the ID experts approached coding. Further evaluations will assess the patients discussed and may consider further evaluation of coding practices used for telehealth.

**Disclosures:**

**Daniel J. Livorsi, MD**, Merck: Grant/Research Support **Robin L. Jump, MD, PhD**, Merck: Grant/Research Support|Pfizer: Advisor/Consultant|Pfizer: Grant/Research Support

